# Predictors of serum dioxin levels among adolescent boys in Chapaevsk, Russia: A cross-sectional pilot study

**DOI:** 10.1186/1476-069X-4-8

**Published:** 2005-05-26

**Authors:** Russ Hauser, Paige Williams, Larisa Altshul, Susan Korrick, Lynne Peeples, Donald G Patterson, Wayman E Turner, Mary M Lee, Boris Revich, Oleg Sergeyev

**Affiliations:** 1 Occupational Health Program, Department of Environmental Health, Harvard School of Public Health, 665 Huntington Avenue, I-1405, Boston, MA 02115, USA; 2 Department of Biostatistics, Harvard School of Public Health, 665 Huntington Avenue, I-415, Boston, MA 02115, USA; 3 Occupational Health Program, Department of Environmental Health, Harvard School of Public Health, 665 Huntington Avenue, I-B26, Boston, MA 02115, USA; 4 Department of Medicine, Brigham and Women's Hospital, Harvard Medical School, Channing Laboratory 336, 11 Longwood Avenue, Boston, MA 02115, USA; 5 Center for Disease Control and Prevention, Toxicology Branch Mailstop F-17, TOX/EHLS/NCEH/CDC, 4770 Buford Hwy NE, Atlanta, GA 30341, USA; 6 Pediatric Endocrine Division, Department of Pediatrics, University of Massachusetts Medical School, 55 Lake Avenue North, Worcester, MA 01532, USA; 7 Center for Demography and Human Ecology of Institute for Forecasting, Russian Academy of Sciences, RAS. 47 Nahimowski Avenue, Moscow 117418, Russia; 8 Chapaevsk Medical Association, Lenina Str., 54B, Chapaevsk, Samara reg. 446100, Russia

## Abstract

**Background:**

Toxicological studies and limited human studies have demonstrated associations between exposure to polychlorinated dibenzo-*p*-dioxins (PCDDs), polychlorinated dibenzofurans (PCDFs) and polychlorinated biphenyls (PCBs) and adverse developmental and reproductive health effects. Given that children may be particularly susceptible to reproductive and developmental effects of organochlorines, and the paucity of information available regarding childhood exposures to dioxins in particular, we undertook a pilot study to describe the distribution of, and identify potential predictors of exposure to, dioxin-like compounds and dioxins among adolescent boys in Chapaevsk, Russia. The pilot study was also designed to guide the development of a large prospective cohort study on the relationship of exposure to PCDDs, PCDFs, and PCBs with growth and pubertal development in peri-pubertal Chapaevsk boys.

**Methods:**

221 boys age 14 to 17 participated in the pilot study. Each of the boys, with his mother, was asked to complete a nurse-administered detailed questionnaire on medical history, diet, and lifestyle. The diet questions were used to measure the current and lifetime consumption of locally grown or raised foods. Blood samples from 30 of these boys were sent to the Centers for Disease Control and Prevention (CDC) for analysis of dioxins, furans and PCBs.

**Results:**

The median (25^th^, 75^th ^percentile) concentrations for total PCDDs, PCDFs and coplanar PCBs were 95.8 pg/g lipids (40.9, 144), 33.9 pg/g lipids (20.4, 61.8), and 120 pg/g lipids (77.6, 157), respectively. For WHO-TEQs, the median (25^th^, 75^th ^percentile) for total PCDDs, PCDFs, and coplanar PCBs were 0.29 (0.1, 9.14), 7.98 (5.27, 12.3), and 7.39 (4.51, 11.9), respectively. Although TCDD was largely non-detectable, two boys had high TCDD levels (17.9 and 21.7 pg/g lipid). Higher serum levels of sum of dioxin-like compounds and sum of dioxin TEQs were positively associated with increased age, consumption of fish, local meats other than chicken, PCB 118, and inversely with weeks of gestation.

**Conclusion:**

The total TEQs among Chapaevsk adolescents were higher than most values previously reported in non-occupationally exposed populations of comparable or even older ages. Dietary consumption of local foods, as well as age and weeks of gestation, predicted dioxin exposure in this population.

## Background

Polychlorinated dibenzo-*p*-dioxins (PCDDs), polychlorinated dibenzofurans (PCDFs), and polychlorinated biphenyls (PCBs) are persistent, lipophilic, halogenated aromatic chemicals that are developmental and male reproductive toxicants in laboratory animals [1-3]. These persistent chlorinated compounds are worldwide environmental pollutants that have been detected in areas as remote as the Arctic [4]. They are biologically concentrated and stored in human adipose tissue with prolonged half-lives. Current estimates of the half-life for 2,3,7,8-tetrachlorodibenzo-*p*-dioxin (2,3,7,8 TCDD) range from 3 to 10 years depending on age, gender, and serum concentration, with faster elimination in men, younger individuals, and those with higher peak exposures [5]. The general population is primarily exposed to these compounds through ingestion of contaminated food (fish, meat, milk, and their by-products), as well as through water sources, dermal contact with soil and house dust, and inhalation [6].

Human studies on the relationship of in utero and childhood (peri-pubertal) exposure to dioxin-like compounds (furans and PCBs) with growth and sexual development in boys are limited. A cross-sectional study of Belgian teenagers (15.8–19.6-year-olds) living in polluted and non-polluted communities demonstrated earlier male pubertal development (assessed by Tanner pubic hair and genital staging) in association with both living in polluted areas and higher serum PCBs as compared to boys living in clean areas and/or with lower PCB levels [7]. Lower testicular volume was associated with living in a polluted area but not with serum PCBs. Dioxin exposure assessed with a bioassay (CALUX) was not associated with male pubertal development in this study.

Several additional epidemiologic studies have been conducted to investigate growth and development in relation to halogenated aromatic compounds. For example, higher prenatal exposure to DDE has been associated with greater male height and weight adjusted for height at puberty and increased weight for height in peri-pubertal girls [8]. In Taiwan, boys exposed *in utero *to PCBs and PCDFs from maternal ingestion of contaminated rice oil had a shorter penile length than unexposed children [9].

Chapaevsk, Russia, is a town of approximately 83,000 residents, located in central Russia (1200 kilometers south-east of Moscow) on the bank of the Chapaevsk river, a tributary to the Volga. The town occupies an area of 187 km^2^, half of which is occupied by industries that are mostly of the military-industrial complex. In 1989, these industries were responsible for the production of the vast majority of the manufactured products from Chapaevsk, and almost half of the city's work force was employed there.

One of the largest chemical factories in Chapaevsk is the Khimprom Chemical Plant (Middle Volga chemical plant), which before 1949 produced chemical warfare agents (such as lewisite and mustard gas). After 1949, there was a transition to the production of industrial and agricultural chemicals, such as gamma-hexachlorocyclohexane (lindane), and other chlorine-containing products such as liquid chlorine, dichloropropionic acid, methyl chloroform, vinyl chloride, and pentachlorophenol [10]. These processes produced PCDDs and PCDFs as industrial contaminants, which subsequently polluted the air, soil, water and food supply in the city [10-12]. Revich and coworkers [10] have found elevated levels of dioxin-like compounds in soil less than two kilometers from the Khimprom Chemical Plant (141 ng TEQ/kg).

The plant has reduced production of these chemicals since 1991; however, since PCDDs and PCDFs persist in the environment, continued human exposure from contaminated air, soil, drinking water, as well as consumption of locally grown vegetables and locally raised animals remains a concern [10-12]. Moreover, a large proportion of the population lives in close proximity to the Khimprom complex.

Given that children may be particularly susceptible to reproductive and developmental effects of organochlorines, and the paucity of information available regarding childhood exposures to dioxins in particular, we undertook a pilot study to describe the distribution of, and identify potential predictors of exposure to, dioxin-like compounds and dioxins among adolescent boys in Chapaevsk, Russia. The pilot study was also designed to guide the development of a large prospective cohort study on the relationship of exposure to PCDDs, PCDFs, and PCBs with growth and pubertal development in peri-pubertal Chapaevsk boys.

## Methods

### Informed Consent

The study was approved by the Human Studies Institutional Review Boards of the Chapaevsk Medical Association, Harvard School of Public Health, University of Massachusetts Medical School, Brigham and Women's Hospital, and Centers for Disease Control and Prevention (CDC). All subjects signed informed consent prior to blood draw and study participation.

### Study Population

From 2579 boys, aged 10–16 years in 1999, enrolled in an earlier pilot study to generate growth and maturation curves for boys in Chapaevsk [13], a subset of 246 older boys (14.0 to 16.9 years) were identified for a sub-study in which blood samples and questionnaire information were obtained. Older boys were chosen for study because blood samples were required and participation rates were expected to be higher than among younger children. Of the 246 boys, 221 had blood samples collected, and of these samples, 30 bloods were initially sent to the CDC for chemical analysis of dioxins, furans and PCBs. By design, of the 30 blood samples, 15 were from children with cryptorchidism or hypospadias, and 15 were from children with neither condition (controls). The selection of the 15 cases and 15 controls was done blindly in relation to factors that may predict dioxin levels.

Each of the 30 boys, with his mother, was asked to complete a nurse-administered detailed questionnaire on medical history, diet, and lifestyle. The diet questions were used to measure the current and lifetime consumption of locally grown or raised foods. The question was worded, "Does your child eat any of the following foods from local sources (i.e. your own garden or farms or lakes in the Chapaevsk area)? Yes/No". There were separate questions for current intake and lifetime intake of each food item. The distances the boys lived from the Khimprom factory at the time of the study and during pregnancy were assessed by questionnaire based on maternal self-report as <2, 2–6, or >6 kilometers, and the distance at the time of the study was also estimated using ArcView GIS 3.0 mapping of addresses.

### Dioxin, furan and PCB chemical analysis

After blood collection and serum separation, the serum samples were stored at -20°C until shipment on dry ice to the US. The chemical analysis was performed by the laboratory at the National Center for Environmental Health, Centers for Disease Control and Prevention (NCEH, CDC), Atlanta, GA. Target analytes included polychlorinated dibenzo-p-dioxins (PCDDs), polychlorinated dibenzofurans (PCDFs), non-ortho substituted (coplanar) polychlorinated biphenyls (co-PCBs), mono-ortho substituted PCBs and other PCBs (nondioxin-like PCBs). Serum samples were spiked with a mixture of ^13^C_12_- labeled PCDDs/PCDFs and coplanar PCBs as internal standards and the analytes were isolated from serum by a C_18 _solid phase extraction (SPE) followed by a multi-column automated cleanup and enrichment procedure [14]. Samples were processed in batches of 10, which included a method blank and two quality control samples that were aliquots from pooled bovine sera spiked with PCDDs, PCDFs and co-PCBs.

The analytes were separated on a DB-5 MS capillary column and quantified using selected-ion-monitoring (SIM) high resolution (10,000 resolving power) mass spectrometry (HRGC-ID/HRMS) by the method described by Patterson et al. [15].

Quantification was by isotope dilution mass spectrometry using calibration standards containing ^13^C labeled, and unlabeled analytes. For specific PCDDs, PCDFs, and co-PCBs that lack their own labeled standard, a labeled congener with the same degree of substitution and a similar retention time was used. Mono-ortho and nondioxin-like PCBs were extracted from an aliquot (1 gram) of sample by SPE extraction [14]. Total Lipids were determined for each serum sample using a "summation" enzymatic method, which is based on the individual measurements of the four lipid groups (free or non-esterified cholesterol, cholesterol esters, triglycerides and phospholipids) [16].

The limits of detection (LODs) were calculated based on long-term standard deviations that were estimated from multiple measurements of low-level standards [17]. For an analyte with a positive blank, the average blank observed during the study was subtracted from individual calculated results [18]. The lipid adjusted detection limits (DLs) were based on observed LODs and corrected for individual sample weight and recovery.

Since values below the DL are expected to have greater variability and uncertainty than values above the DL, some have recommended censoring such data. In the case of our serum analyses, a substantial proportion of our results would be censored at the DL. However, when analyzing group data (as opposed to individual data points), censoring data below the detection limit may lead to the loss of useful information [19]. Where comparisons between uncensored and censored datasets have been performed, the use of DL cut-offs compromised the statistical power of analyses and led to biased exposure estimates [20]. This bias was a greater limitation than any increased variability of low values [18,21]. Thus, in the present study, we retained measurable values below the DL and assigned a value of zero only to samples in which the analyte was not detectable or was at or below the level of contamination in procedural blanks.

The concentrations of PCDDs, PCDFs and co-PCBs were reported on a lipid-adjusted basis as picograms of analyte per gram of lipids (ppt). The concentrations of mono-ortho and nondioxin-like PCBs were reported as nanogram of analyte per gram of lipids (ppb). Toxic equivalents (TEQs) were also reported for PCDDs, PCDFs, co-PCBs and other 'dioxin-like' PCBs based on Toxic Equivalency Factors (TEFs) for individual congeners as specified in the 1998 World Health Organization (WHO) system [22].

### Statistical Analysis

Wilcoxon ranksum tests were used to compare dioxin and PCB levels between controls and boys with cryptorchidism or hypospadias. Generalized linear models were used to identify predictors of the log (base 10) of the sum of dioxin concentrations (PCDDs + PCDFs + co-PCBs) and log sum of dioxin TEQs (PCDDs + PCDFs + Co-PCBs) for the 30 boys. Predictors of dioxin concentrations were first modeled univariately and then adjusted for age. Univariate predictors with p-values below 0.15 were considered for inclusion in the multivariate models. Multivariate model selection was based on adjusted R-square values, Mallows C(p) criterion, and forward and backwards selection procedures; final models used a significance level of 0.05 for inclusion of predictors. Two final models were selected: one which considered all potential covariates, and a second model which considered only those covariates collected on all pilot study participants (i.e., excluding PCB measurements), in order to predict dioxin levels in the larger pilot study cohort.

Dietary exposures were coded as "Yes" or "No" for lifetime consumption of food from local sources (own garden or livestock, friend's garden or livestock, or grown/obtained in Chapaevsk) within the following categories: (1) fruits and vegetables; (2) chicken; (3) non-chicken meats which included goats, cows, pigs, and other non-chicken meat; (4) milk and dairy products, including eggs; and (5) fish. Exploratory models included factors considered a priori to have a potential relationship with serum dioxin levels such as the child's age, body mass index (weight in kg/height in m^2^), and local food consumption habits; reproductive factors (gestational age at birth of the index son, maternal parity [number of live births prior to the index son], gravidity [number of prior pregnancies], and breastfeeding history [cumulative number of weeks of breastfeeding previous children]), residential information (duration in years that the index boy resided in Chapaevsk and distance that mother lived from the factory during pregnancy with the index son and at the time of the study), and socio-demographic factors including parental education, parental occupation, and monthly household income (1 = 300–1000 rubles [$10–$35 U.S.], 2 = 1000–1500 rubles [$35–$50 U.S.], 3 = >1500 rubles [>$50 U.S.]).

## Results

Per our selection criteria, of the 30 boys with serum samples analyzed for dioxin-like compounds, fifteen boys had congenital abnormalities (six hypospadias and nine cryptorchidism). No boy had both malformations. The 15 control boys did not have either hypospadias or cryptorchidism. The distances the boys lived from the Khimprom factory at the time of the study, along with other demographic characteristics, are presented in Table 1. The mean (sd) distance the boys lived from the Khimprom factories was 4.45 (1.76) km. During the pregnancy with her son, of the 29 mothers, 8 (27%) lived less than 2 km, 8 (27%) lived 2–6 km, and14 (45%) lived greater than 6 km from the Khimpron factory. One subject was missing questionnaire data, including distance from Khimprom, medical history, and local food consumption. Only three of the boys' fathers worked at the Khimprom factory during the year prior to the child's birth. While pregnant, two of the boys' mothers reported alcohol intake and one smoked.

**Table 1:** Demographic characteristics of adolescent boys from Chapaevsk, Russia (N = 30)

	**Mean**	**SD**	**N**	**%**
**Age (years)**	16.1	0.81		
**Body Mass Index (kg/m^2^)**	19.9	3.09		
**Weeks of Gestation**^a^	39.2	2.37		
**Breastfeeding duration of index son (weeks)**^b^	36.9	25.2		
**Prior maternal breastfeeding (weeks)**^b^	18.1	38.3		
**Birth weight (gm)**^c^	3433	439		
**Current residential distance to Khimprom factory (km)**	4.45	1.76		
**Time boy resided in Chapaevsk(yrs)**	14.0	3.70		
				
**Gravidity**^d^				
**0**			14	48
**1**			8	28
**2–3**			7	24
**Parity**^d^				
**0**			17	59
**1**			10	34
**2–3**			2	7
**Monthly family income level**				
**300–1000 rubles ($10–$35 U.S.)**			8	28
**1000–1500 rubles ($35–$50 U.S.)**			7	24
**>1500 rubles (> $50 U.S.)**			14	48
**Maximum parental education level**				
**8–11**^th^**grade**			2	7
**Junior College**			17	59
**University**			8	28

^a^One subject was missing data on weeks of gestation of the index son.

^b^One subject was missing data on breastfeeding. Prior maternal breastfeeding is the cumulative number of weeks of breastfeeding children born prior to the index son.

^c^Two subjects were missing data on birth weight of the index son.

^d ^Maternal gravidity is the number of pregnancies prior to the index son and parity is the number of live births prior to the index son.

Since the PCDD, PCDF and PCB distributions were skewed, percentile distributions for serum concentrations and WHO-TEQs for 7 PCDD congeners, 9 PCDF congeners, 3 non-ortho (coplanar) PCBs and 6 mono-ortho PCBs are shown in Table 2. The median (25^th^, 75^th ^percentile) concentrations for total PCDDs, PCDFs and coplanar PCBs were 95.8 pg/g lipids (40.9, 144), 33.9 pg/g lipids (20.4, 61.8), and 120 pg/g lipids (77.6, 157), respectively. The median (25^th^, 75^th ^percentile) concentration for total mono-ortho PCBs was 47.7 ng/g lipids (39.2, 78.4); as expected more than two orders of magnitude larger than for the other compounds. For WHO-TEQs, the median (25^th^, 75^th ^percentile) for total PCDDs, PCDFs, and coplanar PCBs were 0.29 (0.1, 9.14), 7.98 (5.27, 12.3), and 7.39 (4.51, 11.9), respectively. For total mono-ortho PCBs, the median (25^th^, 75^th ^percentile) WHO-TEQ was 8.80 (7.16, 15.5). The total WHO-TEQ for dioxins, furans and PCBs had a median (25^th^, 75^th ^percentile) of 30.9 (18.4, 46.8).

**Table 2:** Distribution of concentrations and WHO-TEQs of PCDD/Fs and PCBs in adolescent boys from Chapaevsk, Russia

**Congener**	**Average DL**	**# of samples above DL**^a^	**Concentrations**			**WHO-TEF**	**WHO-TEQ (pg TEQ/g lipid)**		
			**Percentiles**				**Percentiles**		
			**25-th**	**Median**	**75-th**		**25-th**	**Median**	**75-th**
***PCDDs ***(*pg/g lipid*)									
2,3,7,8-TCDD	4.1	2/29	0.00	0.00	0.00	1.000	0.00	0.00	0.00
1,2,3,7,8-PeCDD	4.8	12/30	0.00	0.00	8.30	1.000	0.00	0.00	8.30
1,2,3,6,7,8-HxCDD	7.1	1/30	0.00	0.00	0.00	0.100	0.00	0.00	0.00
1,2,3,7,8,9-HxCDD	6.9	3/30	0.00	0.00	0.00	0.100	0.00	0.00	0.00
1,2,3,4,6,7,8-HpCDD	14.6	15/30	9.40	14.9	25.1	0.010	0.09	0.15	0.25
1,2,3,4,6,7,9-HpCDD	9.0	0/30	0.00	0.20	1.70	0.0000	0.00	0.00	0.00
OCDD	73.1	15/29	32.9	75.0	100	0.0001	0.00	0.01	0.01
***PCDFs ***(*pg/g lipid*)									
2,3,7,8-TCDF	3.9	0/30	0.00	0.00	0.00	0.1000	0.00	0.00	0.00
1,2,3,7,8-PeCDF	4.7	1/30	0.00	0.00	0.00	0.050	0.00	0.00	0.00
2,3,4,7,8-PeCDF	4.5	29/30	8.50	12.6	17.4	0.500	4.25	6.30	8.75
1,2,3,4,7,8-HxCDF	4.2	27/30	5.50	9.15	20.1	0.100	0.55	0.92	2.01
1,2,3,6,7,8-HxCDF	4.5	21/30	0.00	5.70	8.70	0.100	0.00	0.57	0.87
1,2,3,7,8,9-HxCDF	4.2	0/30	0.00	0.00	0.00	0.100	0.00	0.00	0.00
2,3,4,6,7,8-HxCDF	4.5	1/30	0.00	0.00	1.80	0.100	0.00	0.00	0.18
1,2,3,4,6,7,8-HpCDF	16.8	0/30	3.00	5.30	10.5	0.010	0.03	0.05	0.11
1,2,3,4,7,8,9-HpCDF	6.0	0/30	0.00	0.00	0.00	0.010	0.00	0.00	0.00
***Coplanar PCBs ***(*pg/g lipid*)									
3,4,4',5-TCB 81	11.0	2/30	0.00	0.00	1.60	0.0001	0.00	0.00	0.00
3,3',4,4',5-PeCB 126	25.3	30/30	43.3	68.4	116	0.1000	4.33	6.84	11.6
3,3',4,4',5,5'-HxCB 169	7.7	28/30	27.3	44.2	54.3	0.0100	0.27	0.44	0.54
***Mono-ortho PCBs ***(*ng/g lipid*)									
2,3,3',4,4'-PeCB (105)	11.0	9/27	5.6	7.1	11.8	0.0001	0.56	0.71	1.18
2,3',4,4',5-PeCB (118)	11.0	27/27	20.7	28.0	51.6	0.0001	2.07	2.80	5.16
2,3,3',4,4',5-HxCB (156)	11.0	10/27	7.5	9.1	15.9	0.0005	3.75	4.55	7.95
2,3,3',4,4',5'-HxCB (157)	11.0	0/27	2.3	2.6	4.3	0.0005	1.15	1.30	2.15
2,3',4,4',5,5'-HxCB (167)	11.0	0/27	1.6	2.0	3.7	0.00001	0.02	0.03	0.04
2,3,3',4,4',5,5'-HpCB (189)	10.9	0/25	0.4	0.6	0.8	0.0001	0.04	0.06	0.08
*Total PCDDs (pg/g lipid)*			40.9	95.8	144		0.10	0.29	9.14
*Total PCDFs (pg/g lipid)*			20.4	33.9	61.8		5.27	7.98	12.3
*Total coplanar PCBs (pg/g lipid)*			77.6	120	157		4.51	7.39	11.9
*Total PCDD/F/coplanar PCBs (pg/g lipid)*			154	273	397		12.1	17.1	38.1
*Total Mono-ortho PCBs *(ng/g lipid)			39.2	47.7	78.4		7.16	8.80	15.5
*Total TEQs*								30.9	46.8

Abbreviations: DL, detection limit.

Note: 1,2,3,4,7,8-HxCDD, OCDF, and 3,3',4,4'-TCB 77 were not reported in the present study due to interferences in method blanks.

^a^Results for some samples could not be reported because one or more of the analytical QA/QC criteria were not met. Therefore, the denominator may be less than 30.

Although means and standard deviations do not appropriately describe skewed distributions, to allow for comparisons with other studies we briefly present them. The mean (sd) of total PCDDs, PCDFs, and coplanar PCBs were 104 pg/g lipids (78.0), 63.8 pg/g lipid (89.9) and 123 pg/g lipid (53.1), respectively. The mean of the TEQs for PCDDs, PCDFs, and coplanar PCBs were 5.51, 13.9 and 8.35 pg TEQ/g lipid, respectively.

PCDDs, PCDFs, and coplanar PCBs accounted for a mean percent of total serum dioxin concentrations of 34.3% (range 8.4 to 80.1%), 18.0% (range 4.4 to 57.5%), and 47.7% (range 15.5 to 73.9%), respectively. The relatively high concentrations of mono-ortho PCBs contributed significantly to TEQs and were therefore also included in the total TEQs. PCDDs, PCDFs, coplanar PCBs, and mono-ortho PCBs accounted for a mean percent of total TEQs of 11.9% (range 0.14 to 59.0%), 30.4% (range 3.9 to 71.3%), 25% (range 8.1 to 43.0%), and 32.8% (range 0 to 60.1%), respectively.

**Figure 1:**
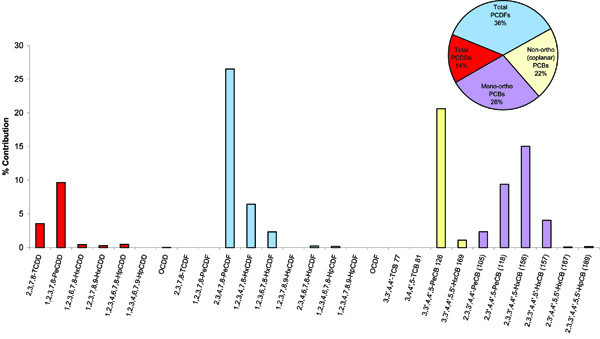
Percent contribution of PCDDFs and PCBs to TEQs (pg TEQ/g lipid) among boys from Chapaevsk.

Figure 1 displays the percent contribution of each PCDD, PCDF and PCB congener to the total TEQ/g lipid for the thirty boys. Based on serum concentrations, the predominant dibenzo-*p*-dioxin congeners were OCDD and 1,2,3,4,6,7,8-HpCDD (mean percents of 73.8% and 22.2%, respectively), while 1,2,3,7,8-PeCDD and 1,2,3,4,6,7,8-HpCDD were the predominant congeners based on mean TEQs (mean percents of 56.2% and 37.0%, respectively). For PCDFs, 2,3,4,7,8-PeCDF and 1,2,3,4,7,8-HxCDF accounted for the majority of the total PCDF concentrations (means of 35.5% and 32.4%, respectively), and these same two congeners accounted for the majority of the total PCDF TEQs (75.2% and 16.7%, respectively). For coplanar PCBs, 3,3',4,4',5-PeCB (#126) and 3,3',4,4',5,5'-HxCB (#169) accounted for the majority of the total coplanar PCB concentrations (means of 63.4 and 35.3%, respectively), and 3,3',4,4',5-PeCB (#126) accounted for 94.1% of the total coplanar PCB TEQs. For mono-ortho PCBs, 2,3',4,4',5-PeCB (#118) and 2,3,3',4,4',5-HxCB (#156) accounted for the majority of the total mono-ortho PCB concentrations (means of 56.0% and 19.7%, respectively), while 2,3,3',4,4',5-HxCB (#156) accounted for 48.3% of the total mono-ortho PCB TEQs and 2,3',4,4',5-PeCB (#118) for 30.3%. The concentrations of six prevalent PCB congeners, or so-called indicator PCBs [23], are presented in Table 3. Here the largest contributors to the sum of the 6 congeners were PCB 153 (34.6%) and PCB138 (20.4%).

**Table 3:** Concentrations (ng/g lipid) of six PCB congeners^a ^in blood of adolescent boys from Chapaevsk, Russia

**PCB congener (IUPAC #)**	**Average DL**	**# of Samples above the DL**^b^	**Median Concentration (ng/g lipid)**	**Mean (SD) Concentration (ng/g lipid)**
PCB 28	20.0	1/24	10.6	11.9 (6.33)
PCB 74	11.0	14/27	10.8	14.8 (10.8)
PCB 99	11.0	27/27	27.7	34.1 (22.5)
PCB 138	18.0	21/27	29.6	39.4 (22.2)
PCB 153	19.8	27/27	51.2	64.5 (30.7)
PCB 180	11.0	23/27	17.7	22.8 (14.9)
Sum of 6 congeners			136	168 (99.3)

Abbreviations: DL, detection limit

^a^PCB congeners include 6 prevalent or indicator congeners (DIN 51 527, 1987)

^b^Results for some samples could not be reported because one or more of the analytical QA/QC criteria were not met. Therefore, the denominator may be less than 30.

Only two of the 30 boys had levels of 2,3,7,8-TCDD greater than the detection limits; levels for these two boys were 17.9 and 21.7 pg/g lipid. Because all samples had different weights (4.1 to 12.1 g), different percent lipids, and percent recoveries, the DLs for TCDD in our study samples ranged from 1.5 to 16.5 pg/g lipid. We were unable to identify unique characteristics of the two boys with detectable levels of 2,3,7,8-TCDD. There were no differences in sum of PCDDs, PCDFS, and PCBs between the group of boys with cryptorchidism or hypospadias and the comparison boys. The smallest difference we could detect however, in the sum of dioxin concentrations given the small size of this study is 175 pg/g lipid (with 80% power) or 200 pg/g lipid (with 90% power). In other words, we can only detect differences that are larger than the observed standard deviation in sum of dioxin-like compounds.

Of the 29 boys with lifetime food consumption data, 86% (n = 25) had consumed local meat products (in particular, 83% had consumed non-chicken meat and 52% had consumed local chicken), 83% (n = 24) had consumed local fish, 93% (n = 27) had consumed locally produced dairy products or eggs, and 97% (n = 28) had consumed locally grown fruits and/or vegetables.

Using regression models adjusting for age, associations were found between the log sum of dioxin concentrations and several characteristics of the thirty boys (Table 4). Increasing age, local non-chicken meat and fish consumption, and log of lipid-adjusted PCB congener 118 were associated with increases in the sum of the dioxin concentrations. For instance, a one- year increase in age increased the mean sum of dioxin-like compounds (291 pg/g lipids) by 30% and consumption of local non-chicken meat increased dioxin-like compounds by 75%. Gestational age in weeks was inversely associated with the sum of the dioxin concentrations, multiplicative factor of 0.92. Although the coefficient for the relationship between log sum of dioxin-like compounds and current residential distance from the Khimprom factory was less than 1.0 (which would support a decrease in dioxin levels with increasing distance from the factory), it was not statistically significant. We also explored the relationship between the sum of dioxin-like compounds and categories of GIS distances from Khimprom plants (<2 km, 2–6 km, >6 km) and found an inverse association, though it too was not statistically significant. Duration of breast-feeding, distance from Khimprom during pregnancy, maternal parity, and parental occupation were unrelated to sum of dioxin-like compounds or sum of dioxin TEQs.

**Table 4:** Individual predictors of log sum of dioxin-like compounds^a^, adjusted for age in years

**Predictor**	**Estimate**	**p-value**	**Multiplicative factor on dioxin (95% CI)**	**R-Square Value**
Age of index son (years)	0.26	0.057	1.30 (1.00–1.72)	0.13
BMI of index son (kg/m^2^)	0.03	0.40	1.03 (0.95–1.13)	0.15
Consumption of local non-chicken meat (y/n)	0.56	0.042	1.75 (1.05–2.92)	0.26
Consumption of local chicken (y/n)	0.13	0.54	1.14 (0.75–1.72)	0.14
Consumption of local fish (y/n)	0.48	0.079	1.62 (0.97–2.71)	0.23
Consumption of local eggs (y/n)	0.20	0.36	1.23 (0.80–1.88)	0.16
Consumption of local dairy (y/n)	-0.19	0.65	0.82 (0.36–1.89)	0.13
Consumption of local fruits/vegetables (y/n)	-0.12	0.84	0.89 (0.28–2.77)	0.13
Current residential distance from Khimprom (km)	-0.06	0.37	0.94 (0.82–1.07)	0.15
Years resided in Chapaevsk	0.009	0.75	1.01 (0.95–1.07)	0.13
Monthly family income level^b^	-0.09	0.50	0.92 (0.71–1.18)	0.14
Maximum parental education^b^	0.21	0.26	1.23 (0.86–1.76)	0.17
Log (PCB 118) (ng/g lipid)	0.64	<0.001	1.90 (1.37–2.63)	0.46
Congenital abnormality of index son	-0.015	0.95	0.99 (0.64–1.51)	0.13
Parity^b,c^	-0.011	0.94	0.99 (0.74–1.32)	0.13
Weeks of gestation	-0.08	0.072	0.92 (0.84–1.00)	0.23
Weeks of breast-feeding index son (per 12 weeks)	-0.004	0.94	1.00 (0.90–1.10)	0.13
Prior breastfeeding duration^d ^(years)	0.09	0.53	1.10 (0.82–1.47)	0.14

Abbreviations: Log, logarithm base 10; BMI, body mass index; PCB 118, polychlorinated biphenyl 118

^a^Sum of dioxin-like compounds includes PCDDs, PCDFs, and coplanar PCBs.

^b^Each variable was modeled using three ordinal levels.

^c^Parity refers to the number of live births prior to the index son.

^d^Prior maternal breastfeeding refers to the cumulative number of weeks of breastfeeding children born prior to the index son.

N = 30 for age, BMI, and distance from Khimprom at time of blood draw; N = 29 for all food consumption and medical history predictors; N = 27 for model with PCB118.

Models predicting the log of the sum of dioxin TEQ's (Table 5) were generally similar in magnitude and direction for the predictors shown in Table 4, but suggested weaker effects of age (multiplicative factor = 1.30, p = 0.14) and local fish consumption (multiplicative factor = 1.43, p = 0.32), and a stronger effect of maximal parental education (multiplicative factor = 1.50, p = 0.086) and log of PCB 118 TEQ (multiplicative factor = 2.61, p < 0.001).

**Table 5:** Predictors of log dioxin TEQs^a ^among boys in Chapaevsk, Russia (adjusted for age).

**Predictor**	**Estimate**	**p-value**	**Multiplicative factor on dioxin (95% CI)**	**R-Square Value**
Age (years)	0.26	0.14	1.30 (0.93–1.81)	0.08
BMI (kg/m2)	0.04	0.41	1.04 (0.95–1.13)	0.10
Consumption of local non-chicken meat (y/n)	0.68	0.059	1.97 (1.01–3.87)	0.20
Consumption of local chicken (y/n)	0.06	0.82	1.07 (0.62–1.83)	0.08
Consumption of local Fish (y/n)	0.36	0.32	1.43 (0.71–2.88)	0.11
Consumption of local Eggs (y/n)	0.12	0.68	1.13 (0.64–1.98)	0.09
Consumption of local Dairy (y/n)	-0.43	0.44	0.65 (0.22–1.89)	0.10
Consumption of local Fruits/Vegetables (y/n)	0.35	0.65	1.41 (0.32–6.17)	0.09
Current residential distance from Khimprom (km)	-0.09	0.32	0.91 (0.77–1.09)	0.12
Years resided in Chapaevsk	-0.014	0.71	0.99 (0.92–1.06)	0.09
Monthly family income level^b^	-0.14	0.41	0.87 (0.63–1.21)	0.10
Maximum parental education^b^	0.41	0.086	1.50 (0.96–2.36)	0.18
log (PCB118 TEQ)	0.96	<0.001	2.61 (1.81–3.75)	0.57
Sexual delay/congenital abnormality	0.21	0.46	1.23 (0.71–2.13)	0.10
Parity^b,c^	0.06	0.77	1.06 (0.73–1.54)	0.09
Weeks of gestation	-0.11	0.078	0.90 (0.80–1.01)	0.19
Breast-feeding duration of index son (per 12 weeks)	-0.002	0.97	1.00 (0.87–1.14)	0.08
Prior breastfeeding duration^d ^(years)	0.07	0.72	1.07 (0.73–1.57)	0.08

Abbreviations: Log, logarithm base 10; BMI, body mass index; TEQ, toxic equivalents

^a^Sum of dioxin TEQs include TEQs for PCDDs, PCDFs, coplanar PCBs and other 'dioxin-like' PCBs

^b^Each variable was modeled using three ordinal levels.

^c^Parity refers to the number of live births prior to the index son.

^d^Prior maternal breastfeeding refers to the cumulative number of weeks of breastfeeding children born prior to the index son.

N = 30 for age, BMI, and distance from Khimprom at time of blood draw; N = 29 for all food consumption and medical history predictors; N = 27 for model with PCB118.

The best fitting multivariate models for predicting the sum of dioxin concentrations are summarized in Table 6. Even after accounting for the strong relationship between PCB 118 concentration and the sum of dioxin concentrations on the log scale, consumption of local non-chicken meat showed a significant association with increased dioxin levels. Similarly, the best fitting multivariate model for predicting the sum of dioxin TEQs also included PCB 118 and consumption of local non-chicken meat (R-square = 0.63). The best-fitting model for sum of dioxin concentrations, excluding log PCB 118, included age, weeks of gestation, income level, and consumption of local non-chicken meat and local dairy products, with an R-square of 0.56. The best fitting multivariate model for predicting the sum of dioxin TEQs, excluding log PCB 118 TEQ, included the same five predictors as those shown in Table 6 with similar magnitudes and significance of effects (R-square = 0.56).

**Table 6:** Multivariate models for predicting log sum of dioxin^a ^concentrations among adolescent boys in Chapaevsk, Russia

**Model #**	**Predictor**	**Estimate**	**p-value**	**R-square value**	**Adjusted R-square value**
1	Consumption of local non-chicken eat (y/n)	0.59	0.017	0.56	0.52
	log (PCB 118) (ng/g lipid)	0.63	<0.001		
2	Age (years)	0.40	<0.001	0.56	0.46
	Weeks of gestation	-0.11	0.005		
	Consumption of local dairy (y/n)	-0.76	0.037		
	Consumption of local non-chicken meat (y/n)	0.90	<0.001		
	Monthly family income level (ordinal, low, med, high)	-0.27	0.020		

Abbreviations: Log, logarithm base 10

^a^Sum of dioxin-like compounds includes PCDDs, PCDFs, and coplanar PCBs

N = 26 for model 1; N = 29 for model 2. Missing data for 3 boys on lipid-adjusted PCB 118, and one boy on questionnaire items (medical history, diet).

Model 1: best fit multivariate model including PCB 118 data.

Model 2: best fit multivariate model (forward and backward selection) not including PCB 118.

## Discussion

Among adolescent boys in Chapaevsk, Russia, higher serum levels of sum of dioxin-like compounds and sum of dioxin TEQs were positively associated with increased age, consumption of fish, local meats other than chicken, and inversely with weeks of gestation. The age association was found despite a narrow age range of slightly over two years in our study. Although not statistically significant, the distance the boy lived from the Khimprom factory at the time of blood draw was inversely associated with serum levels of sum of dioxin-like compounds and sum of dioxin TEQs. As expected, serum PCBs, specifically PCB 118, were strongly associated with both sum of dioxin-like compounds and sum of dioxin TEQs.

There was no association between the distance of the residence from the Khimprom plant during the pregnancy and subsequent serum dioxin levels. One potential explanation for the lack of association may include misclassification of distance since the mother was asked to recall a time period more than 14 years prior to the study. However, we would expect that the mother would be able to recall residential history at the time of the birth of their son. Mother's self-reported estimates of current residential distance from Khimprom was generally accurate; twenty-one of twenty-nine mothers correctly categorized their current residential distance from the Khimprom plants based on cross-referencing using GIS mapping. Other explanations include that prenatal exposure 14 or more years prior to the current serum sample is not as strong a predictor as are exposures resulting from present residential location. Although there was no association of case status (cryptorchidism or hypospadias) with dioxin levels, we did not have sufficient power to definitively assess this relationship.

Perinatal history (e.g. weeks of breastfeeding) was generally not associated with exposure measures in this population; this may be a function of older age of the children. However, there are limited data on the relationship of perinatal factors with organochlorine exposures in this age group so a null finding is of interest given reports that differences in organochlorine levels among breastfed and non-breastfed are generally no longer discernable by early school age [24,25] and, furthermore, that dietary intake after this age contributes significantly to total dioxin intake [26]. In prior studies, substantial emphasis has been placed on pre- and early postnatal (via breastfeeding) exposures because of particular vulnerability during fetal and early infant development. The exposure risk factors during peri-adolescence, another period of potential developmental vulnerability, has not been studied in-detail, therefore, the identification of exposure risk factors specific to this period will enhance our understanding of this critical period.

**Figure 2:**
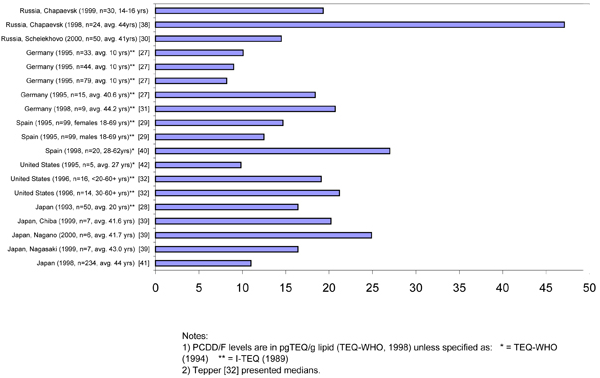
Mean PCDD/PCDFs TEQ levels in Chapaevsk boys in comparison with other populations.

Although data on levels of PCDD/PCDFs in children is limited, our results suggest that the mean total TEQs among Chapaevsk adolescents were higher than most values previously reported in non-occupationally exposed populations of comparable or even older ages. Figure 2 shows a comparison of the mean PCDD/PCDFs TEQ levels in Chapaevsk boys with other populations (TEQ from dioxin-like PCBs was not included, since some of these studies did not report them). The mean TEQs of pooled blood samples from 10 year-old German boys in rural and urban settings was 8.2 pg TEQ/g lipid for an urban industrial area, 9.0 pg TEQ/g lipid for an industrial area within a rural setting, and 10.1 pg TEQ/g lipid for a rural area [27]. In comparison, the mean TEQ in the Chapevsk boys was 19.3 pg TEQ/g lipid. With the exception of children described by Wuthe et al. [27], subjects in the other studies in Figure 2 were significantly older than the Chapaevsk boys. Despite age differences, the mean TEQ in Chapaevsk boys was comparable to or even higher than the mean TEQ in older populations from other countries. For example, they were higher than mean TEQs of 18.4 pg TEQ/g lipid in adults (40.6 years old average) from South Germany [27] or 16.4 pg TEQ/g lipid from 20 year old Japanese women [28] or pooled samples from randomly selected males and females 18–69 years of age from the Spanish city of Mataro [29], which were 12.5 and 14.7 pg TEQ/g lipid respectively. The mean levels in adult female (mean age 41 years) non-factory workers in the Russian city of Shelekhovo were also lower at 14.5 pg TEQ/g lipid [30]. Mean TEQs for the general population (mean age 44.2 years) in Germany, collected in 1997–98 [31], and median TEQs for long-term workers of pulp and paper mill and non-workers in the U.S. in 1996 [32] were similar to levels found in the Chapaevsk boys. The mean levels in the study in Germany were 20.71 pg TEQ/g lipid and in the U.S. the median levels were 19.1 pg TEQ/g lipid for community residents and 21.2 pg TEQ/g lipid for low exposure workers.

Although TCDD was largely below the detection limits in this small pilot sample, the two boys with detectable values had high TCDD levels (17.9 and 21.7 pg/g lipid), suggesting that exposure for at least some portion of this population is substantially higher than typical of this age group. In comparison, in a cohort of adult (mean age of 58 years) fishermen from a polluted region of Finland, the mean TCDD concentration was 19 pg/g lipid [33]. In adult (mean age of 53 years) residents from Calcasieu Parish, Louisiana, which is near a chemical industrial complex, the mean TCDD level was 7.6 pg/g lipid [34]. Not only were the dioxin levels in these two children higher than those found in these studies, but the adults in the previous studies were several decades older and therefore would be expected to have higher dioxin body burdens than younger children [35]. Potential explanations for the large number of non-detectable samples for TCDD include the small sample volume and young age of the subjects. In our future studies in this population, we will collect larger volumes of serum for dioxin analysis.

In one of the few studies on dioxin-like compounds in which children were included, Eskenazi and coworkers [36] evaluated the relationship between serum TCDD concentrations and age at exposure of female residents of Seveso, Italy. Residents near the ICMESA chemical plant in Seveso were exposed to some of the highest known residential levels of 2,3,7,8-TCDD as a result of an explosion at the plant. Archived serum collected near the time of the accident was used to measure exposures. Residents closest to the plant had a median 2,3,7,8-TCDD level of 272 ppt (IQR 92 – 883 ppt). Residential proximity to the plant and younger age (up to 13 years old) were the strongest predictors of an individual's serum 2,3,7,8-TCDD level. Other predictors included being outdoors at the time of explosion and consumption of homegrown food. The higher levels found in children were most likely a result of increased exposure as a result of activity patterns and a greater proportionate consumption of food, water and air than adults [37].

Although the exposure scenario (an acute high exposure event) is different than the chronic low/moderate exposure occurring in Chapaevsk, the results from Seveso suggest that children may be at increased risk for high dioxin exposure from environmental contamination. In our study, although distance from the Khimprom plants was a weak predictor of serum dioxin-like compounds, consumption of local foods (specifically meat and fish), as in the Seveso study, was a strong predictor of sum of dioxin-like compounds and dioxin TEQs. This finding is notable given concerns regarding environmental dioxin contamination in the community and suggests that food may be one of the more, if not most, important routes of environmental contaminant exposure for residents in this setting. In other settings, contaminated food generally contributes much more substantially to human organochlorine burden than air or soil (which may be related to residential proximity to pollutant sources) [6]. We will investigate this issue in more detail in our ongoing study.

## Conclusion

Recently, data were published on dioxin levels among adult residents of Chapaevsk, Russia [38]. Twenty-four self-selected volunteers (12 men and 12 women) provided blood samples in 1998 for analysis of dioxin-like compounds. These serum samples were analyzed by the same laboratory at the CDC, which analyzed the serum samples for our study. None of the adult subjects worked at the Khimprom plant in Chapaevsk. The mean age of subjects was 44 years for men and 45 years for women. Among all subjects, the mean TEQ of total dioxins was 61.2 pg TEQ/g lipid (range 16.4 to 168.1 pg TEQ/g lipid). The adults had the same congeners as major contributors to TEQ of dioxin, furan and co-planar PCBs as the boys in our study. We could not compare the levels of mono-ortho-PCBs in the children to levels in the adult Chapevsk residents since they were not reported. In the adults in Chapaevsk, there were positive relationships of TEQs with age and BMI and an inverse relationship of TEQs with residential distance from the plant [38]. In both this adult and our pediatric Chapaevsk population, PCB congeners contributed significantly to TEQs and therefore it is important to assess these congeners vis-à-vis toxicological implications mediated through dioxin-like mechanisms.

We have recently begun recruiting eight- and nine-year old boys and their families in Chapaevsk into a prospective cohort study on the relationship between dioxin exposure and somatic growth and pubertal development. Results of this exposure assessment study demonstrate relatively high TEQ exposures among Chapaevsk children compared to similar aged populations elsewhere and, as evidenced by the importance of local food consumption in determining this exposure, corroborate our hypothesis that local environmental contamination likely contributes to exposure risk in this setting. Our planned additional studies of the potential health consequences of Chapaevsk children's exposure is an important sequel to having characterized organochlorine exposure and exposure risk among this potentially vulnerable age group.

## List of Abbreviations

IUPAC International Union of Pure and Applied Chemistry

mono-*ortho *PCBs Includes PCB congener numbers 105, 114, 118, 123, 156, 157, 167, 189

non-*ortho *PCBs Includes PCB congener numbers 77, 81, 126, 169

OCDD Octachlorodibenzo-*p-*dioxin

OCDF Octachlorodibenzo-furan

PCBs Polychlorinated biphenyls

PCDDs Polychlorinated dibenzo-p-dioxins

PCDFs Polychlorinated dibenzofurans

pg/g Picogram per gram, 10^-12 ^g.

TEF Toxic equivalency factors

TEQ Toxic Equivalents (WHO_98_)

I-TEQ International Toxic Equivalents (NATO-CCMS, 1988)

WHO World Health Organization

## Competing interests

The author(s) declare that they have no competing interests.

## Authors' contributions

RH conceived the design of the study and took primary responsibility for drafting the manuscript. PW performed the statistical analysis, participated in the design of the study and the drafting of the manuscript. LA participated in the drafting of the manuscript and the interpretation of the dioxin results. SK participated in the design of the study and the drafting of the manuscript. LP participated in the statistical analysis. DGP carried out the dioxin analyses. WET carried out the dioxin analyses. MML participated in the design of the study and the drafting of the manuscript. BR participated in the design of the study. OS oversaw subject recruitment and data collection, participated in the design of the study and the drafting of the manuscript. All authors read and approved the final manuscript.
